# GPU-accelerated visualization of historical wildfire events with temporal geospatial data integration

**DOI:** 10.1016/j.mex.2026.104080

**Published:** 2026-07-30

**Authors:** Rafael José, João Carvalho, Afonso Oliveira, Houda Harkat, Nuno Fachada

**Affiliations:** a Lusófona University, Lisboa, 1749-024, Portugal; b LASIGE, Departamento de Informática, Faculdade de Ciências, Universidade de Lisboa, Lisboa, 1749-016, Portugal; c Center of Technology and Systems (UNINOVA-CTS) and Associated Lab of Intelligent Systems (LASI), Caparica, 2829-516, Portugal; d INESC INOV, Lisboa, 1000-029, Portugal

**Keywords:** Historical wildfire reconstruction, Geodetic coordinate transformation, Wind data integration, Particle-based rendering, Object pooling, Real-time rendering

## Abstract

Wildfires severely affect ecosystems and populations. Reconstructing documented fires helps emergency services study hazards and train responders. Many current tools are difficult to integrate and lack real-time performance across scenarios. This paper presents a GPU-accelerated Unity system for replaying and rendering documented wildfire events with temporal precision. The proposed method is a visualization pipeline for historical fire records—not a fire propagation model—using recorded rather than computed ignition and extinction times. It integrates geospatial processing, particle rendering, and temporal event modeling for fire behavior analysis. The system processes historical data chronologically, transforms geographic coordinates to maintain spatial fidelity, and renders fire stages with terrain-aligned particles. It incorporates wind data and applies performance optimizations including object pooling and GPU-accelerated particle rendering. Results illustrate reconstruction of documented fire patterns with stable, real-time frame rates in high-density scenarios, highlighting applicability for scientific analysis and training.

• A geodetic transformation pipeline converting historical fire coordinates to a spatially consistent metric frame using ellipsoidal Earth geometry.

• A deterministic temporal scheduler enabling timestamp-faithful replay of fire sequences with GPU-accelerated particle rendering and meteorological integration.

• Performance validation demonstrating real-time interactive frame rates with up to 407 simultaneous fires on dedicated and integrated GPUs.

## Graphical abstract

**Figure:**
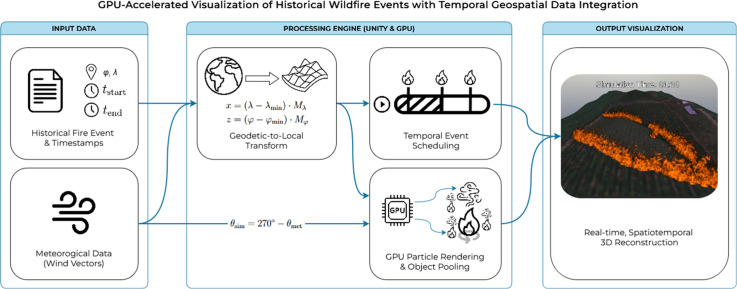

## Specifications table

**Table:** 

**Subject area**	Computer Science
**More specific subject area**	Scientific Visualization; Real-time Rendering; Environmental Simulation
**Name of your method**	GPU-Accelerated Wildfire Visualization Pipeline
**Name and reference of original method**	Not applicable
**Resource availability**	Code, Unity project, datasets, and analysis scripts: https://github.com/rafaeldavidjose/gpu-wildfire-visualization (MIT license)

## Background

Wildfire modeling and visualization are major challenges in computational environmental science and emergency management [1]. Traditional techniques often focus on either computational accuracy or visual realism, rarely both [2], trading timing precision for computational performance [3]. Correct geographic coordinate transformation is essential to faithfully represent observed fire locations and progression, since inconsistencies may distort spatial fire-spread estimates [4]. Incorporating meteorological information also requires careful synchronization for environmental consistency.

To enable real-time, at-scale visualization, Graphics Processing Units (GPUs) are a practical option for environmental simulation [5], providing the parallel computing power needed to overcome the bottlenecks of traditional wildfire visualization systems. Modern GPU architectures efficiently handle the calculations required for geodetically consistent coordinate transformations and dynamic fire behavior rendering [6,7], enabling real-time processing of large-scale datasets.

This article describes a comprehensive wildfire visualization methodology using the Unity game engine [8], defined by the following set of novel capabilities, which to our knowledge have not been offered together in existing systems:

1. A geodetic transformation model which ensures spatial consistency by accounting for Earth's ellipsoidal geometry.
2. A temporal event framework that reconstructs historical fire sequences with precise timing.
3. Integration of meteorological data, especially wind dynamics, driving the visual behavior of rendered fires from recorded conditions.
4. GPU-accelerated performance optimization strategies enabling interactive rendering of large-scale fire datasets.

The proposed method is a replay-and-rendering pipeline for documented fire event sequences: ignition and extinction locations and times are read from recorded data, and wind affects only the appearance of the rendered fires. It targets post-event analysis and training, can serve as a visualization front end for any fire-spread model's output, and does not involve a physical propagation model or predictive use.

Table 1 summarizes representative systems published since 2020 and the capability gap this work addresses: most tools emphasize predictive modeling, aesthetic rendering, or algorithmic simulation without combining (i) an ellipsoidal Earth transformation for geodetic consistency, (ii) deterministic, timestamp-faithful sequencing, (iii) environmental forcing in the renderer, and (iv) interactive performance on large historical datasets. Our framework combines these four properties, enabling chronologically faithful, geodetically consistent, interactive reconstructions. Capability criteria and per-cell justifications are given in the table note.

**Table 1:** Comparison of capabilities in recent wildfire visualization systems. The symbols ✓, ∼, and ✗ indicate full, partial, and no support, respectively. GPU accel.: core computations run on the GPU beyond standard rasterization; Historical recons.: reproduces a documented fire event; Real-time rendering: interactive performance (>30 FPS); Temp. precision: events follow explicit real-world timestamps rather than simulation time steps; Env. integration: environmental data influence the runtime fire state.

System/Reference	GPU accel.	Historical recons.	Real-time rendering	Temp. precision	Env. integration
Fire in Paradise [9]	✓	✗	✓	✗	✓
3D Forest Fire [10]	✓	✗	✓	✗	✓
Patagonia HPC [11]	✓	✓	✓	✗	✓
vFirelib [7]	✓	✗	✓	✗	✓
Weather-based 3D [12]	✗	✗	✓	✗	✓
2D GPU Fire [13]	✓	✗	✗	✗	✓ ^a^
Scintilla [14]	✓	✗	✓	✗ ^b^	✓
Firefighting VR [15]	✗	✗	✓	✗ ^c^	✓
PyTorchFire [16]	✓	∼ ^d^	✗	∼ ^d^	✓
VIIRS Mapping [17]	✓ ^e^	✓	✗	✓ ^f^	✗
Our System	✓	✓	✓	✓	✓

ᵃWind and topography enter the model as a convection field. ᵇ, ᶜ Integration or engine time steps only; no timestamp scheduling. ᵈ Calibrates to observed perimeters; does not replay recorded timestamps. ᵉ GPU-trained deep network; no interactive rendering. ᶠ Tied to acquisition timestamps (12-hour revisit).

The comparison highlights the gap in temporally precise reconstruction of documented events. Only the VIIRS pipeline ties outputs to real-world timestamps, at 12-hour granularity and without interactive rendering [17]; none combines environmental integration with timestamp-driven scheduling and interactive rendering. The proposed method combines these capability axes within a single pipeline, enabling accurate replay of historical wildfires while preserving interactivity for analysis and training.

### Method details

**Fig. 1:**
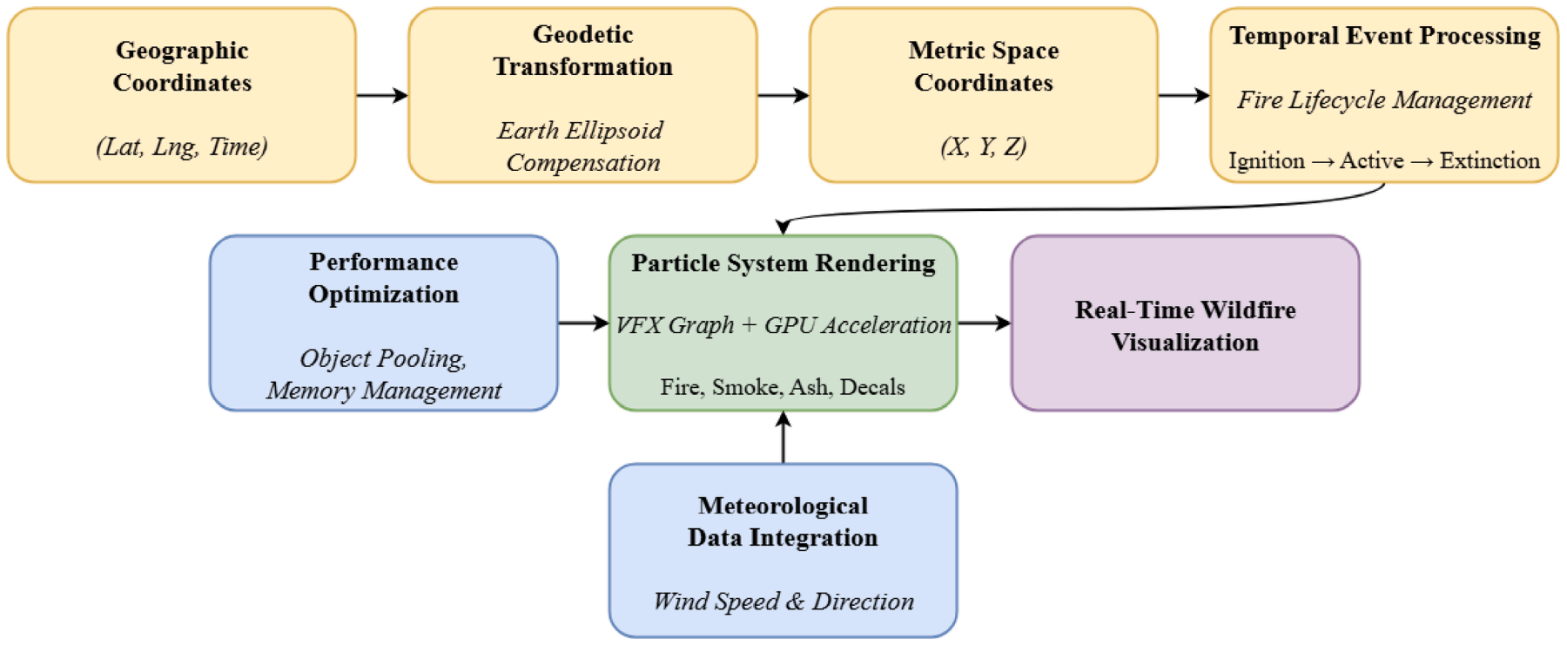
System architecture overview showing the data flow from geographic coordinates through transformation, temporal processing, and particle system rendering.

The system comprises four modules: geospatial processing, temporal event management, meteorological integration, and visual rendering. These modules are connected through standardized interfaces and implemented in Unity, running inside the Unity Editor. Fig. 1 shows the data flow from geographic coordinate ingestion and transformation, through temporal fire-event processing, to particle-system rendering.

The core data model represents each fire as an event record. Fire events are stored in a time-ordered list (sorted by ignition) for single-pass, deterministic replay, with a companion hash map keyed by event ID that matches extinction records to their events when the datasets are merged at load time. Each record includes geographic position, ignition and extinction timestamps, lightweight lifecycle flags, and references to reusable visual assets. This arrangement scales to large datasets while preserving chronological fidelity.

The following subsections detail the modules in Fig. 1: geodetic coordinate transformation, temporal event processing, fire-lifecycle visualization, meteorological integration, and performance optimization.

### Prior work

GPU-based environmental visualization has received growing attention [7,10,12]. Most frameworks target predictive modeling, leaving a gap for systems that reconstruct documented events with strict timing. Wu et al. [7] present vFirelib, a GPU-based tool that computes burn-distance fire spread in parallel. The system uses CUDA for spread calculations and reports substantial speedups over sequential baselines, with visualization serving as a front end to the accelerated simulation. Xia and Cheng [16] introduce PyTorchFire, a differentiable cellular-automata model that enables learning-based spread dynamics. You et al. [10] propose a real-time 3D visualization system that encodes tree morphology with finite-state machines to achieve interactivity. Collectively, these works primarily accelerate fire-spread simulation on GPUs and, in some cases, the rendering pipeline; they inform our renderer but do not target temporally faithful replay of documented events.

Waidelich et al. [18] implement parallel stochastic simulations to visualize probability maps for decision support, with interaction via landscape rotation and layer opacity. Yang et al. [15] build an immersive decision-making framework for resource allocation and firefighting inference, coupling optimization with interactive visualization. Denham et al. [11] develop an HPC simulator for Patagonia focused on large-scale propagation; the visualization is functional but secondary to compute scalability. Overall, these systems emphasize interactivity and real-time responsiveness but primarily serve forecasting or optimization rather than timestamp-faithful reconstruction.

Graphics-oriented fire visualization has advanced visual realism. Hädrich et al. [9] present Fire in Paradise, combining mesoscale simulation with cinematic techniques. Kokosza et al. [14] introduce Scintilla, a fire visualization approach targeting believable appearance and performance. Meng et al. [12] integrates multiple weather factors into a real-time renderer. These systems highlight techniques that improve perceived realism and user engagement, which are relevant to our renderer, yet they typically do not enforce geodetic alignment or timestamp-faithful playback.

Yun et al. [19] integrate OpenSceneGraph with the FARSITE simulator to visualize physics-based propagation, trading ease of integration for fidelity to the simulation core. Ghodrat et al. [20] analyze wind–fire interaction models; their contributions are primarily algorithmic, with visualization used mainly for inspection rather than temporally controlled replay. Satellite-driven and ML-based systems address detection and prediction. Zhao and Ban [17] use autoregressive spatiotemporal models to map burned areas from VIIRS in near real time, focusing on detection rather than chronological visualization. Li et al. [21] implement ML-based prediction in an interactive web map. Shennan et al. [22] couple thermal infrared imagery with web geovisualization for landscape-level analysis. Sun et al. [23] review wildfire spread risk and behavior modeling and note the predominance of predictive tools over temporally precise visualization of historical sequences. Koutsaris [24] combines remote-sensing data with a game engine for geospatial forest visualization in XR.

### Geodetic coordinate transformation

Geodetic-to-local conversion is implemented by linearizing geographic coordinates into a Unity-local metric frame using latitude-dependent meters-per-degree factors. To convert geodetic coordinates (latitude and longitude) into a planar metric coordinate system, the system first computes the linear distances corresponding to one degree of latitude and one degree of longitude at a given geographic location.

Due to the Earth’s ellipsoidal shape, these distances vary with latitude. Here, we denote the *meridional distance per degree of latitude* as $M_{\varphi }$ and the *zonal distance per degree of longitude* as $M_{\lambda }$. These quantities represent the approximate number of meters equivalent to one degree change in latitude and longitude, respectively. The meridional distance $M_{\varphi }$ is calculated by:

$$\begin{array}{c} M_{\varphi }=111132.92-559.82\cos\left(2\varphi \right)\\ \;\;+1.175\cos(4\varphi )-0.0023\cos(6\varphi ) \end{array}$$

where φ is the latitude in radians. This equation accounts for variations in distance per degree due to the Earth’s curvature. Similarly, the zonal distance $M_{\lambda }$, which decreases as the latitude increases, is given by:

$$\begin{array}{c} M_{\lambda }=111412.84\cos\left(\varphi \right)\\ \;\;\;-93.5\cos(3\varphi )+0.118\cos(5\varphi ) \end{array}$$

Using these computed values, the transformation from geodetic to metric coordinates in the simulation is performed as follows:

$$\begin{array}{c} x=\left(\lambda -\lambda_{\text{min}}\right)\cdot M_{\lambda }\\ z=\left(\varphi -\varphi_{\text{min}}\right)\cdot M_{\varphi } \end{array}$$

Here, λ and φ represent the longitude and latitude (in decimal degrees) of a given point, while $\lambda_{\text{min}}$ and $\varphi_{\text{min}}$ define the minimum longitude and latitude boundaries of the simulation domain. The result is a coordinate system in meters that aligns accurately with the Earth’s surface, enabling precise positioning of wildfire events within the visualization environment.

Terrain elevation data are also sampled to ensure that the vertical placement of fire instances is consistent with the ground topography, thereby avoiding visual inconsistencies such as floating or submerged fire representations.

**Fig. 2:**
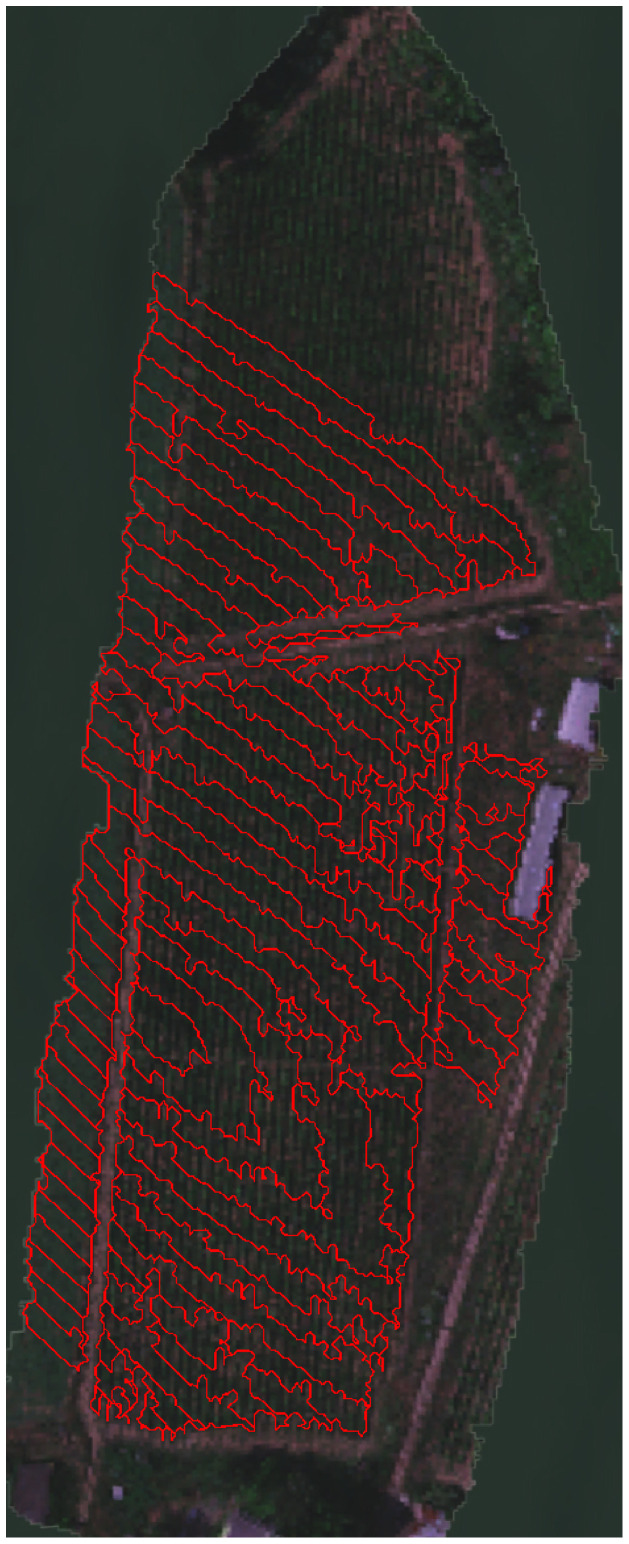
Polygon overlay illustrating registration of the transformed coordinates over a top-down view. The red outline corresponds to the transformed polygon.

The validity of this geodetic transformation approach has been confirmed through polygon overlay tests, which demonstrate consistent registration between the transformed coordinates and known geographic boundaries (Fig. 2), and quantified against double precision geodesic reference positions, with sub-meter displacement for all records (see Coordinate transformation accuracy).

Unlike common projection-based approaches (e.g., the Universal Transverse Mercator [25]), this transformation directly estimates linear distances from latitude and longitude without requiring map projection libraries, simplifying integration with Unity’s local coordinate system. Minor trade-offs in projection fidelity are offset by higher performance and acceptable accuracy for visualization tasks.

### Temporal event processing

Fire events are scheduled by a single-pass time-ordered processor. It advances a simulation clock and triggers ignition or extinction when the corresponding timestamps are reached. The system maintains a list *E* sorted by ignition time and an active set S with currently burning events. A hash map indexed by event ID matches extinction records to their events when the data are loaded. A pointer *i*_next_ scans *E* exactly once, so ignition costs amortized constant time per frame, and each event is activated and extinguished exactly once. In the implementation, *S* is realized through lifecycle flags. The extinction check scans the activated prefix of *E* and acts only on events whose flags mark them as burning. The complete scheduling procedure is given in Algorithm 1.

**Algorithm 1:**
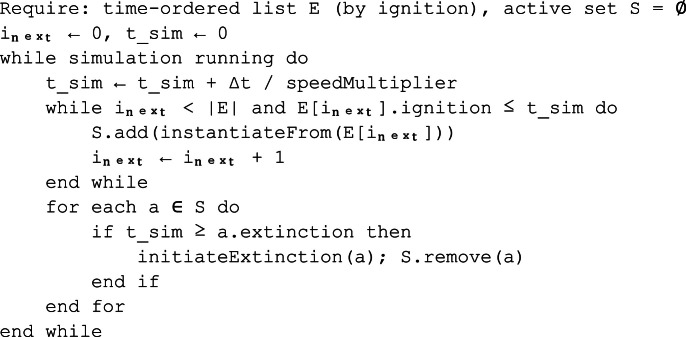
Temporal scheduler for ignition and extinction based on historical timestamps. Parameters: Δt (wall-clock step) and speedMultiplier (time-compression factor).

Ignition and extinction times arrive as strings in the input JSON (e.g., 5H:30 M); the loader parses them into integer minutes for direct comparison with the simulation clock, using $t_{\text{minutes}}=60h+m$. This normalization removes parsing overhead from the scheduler and makes time comparisons frame-rate independent.

### Fire event visualization and lifecycle management

The fire visualization component renders each fire instance (one record of the core data model) across its lifecycle using rendering assets: pooled particle-system instances for flame and smoke, and a terrain-aligned burn-mark decal. Assets are parameterized by the simulation clock and by the wind vector provided by the meteorological component; placement is terrain-accurate via height sampling.

**Fig. 3:**
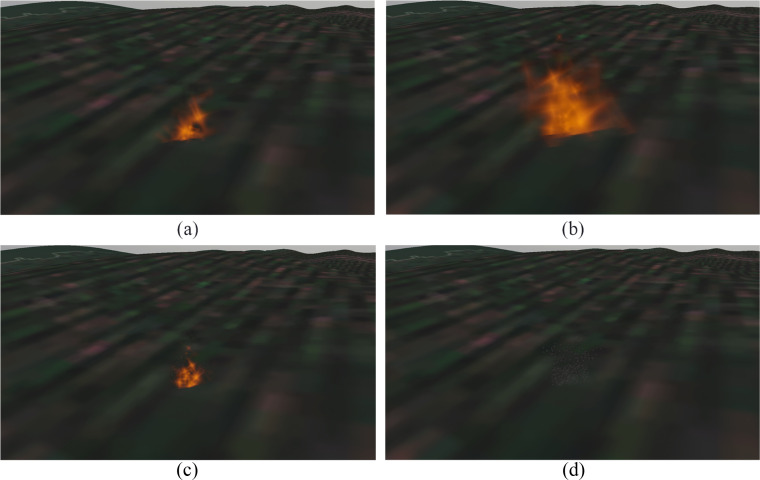
Per-instance fire lifecycle: (a) ignition with ramp-in; (b) burning with wind-oriented emission; (c) extinction with emission ramp-down and ash emergence; (d) residue with persistent terrain decal.

As illustrated in Fig. 3, each instance follows a finite-state lifecycle with four states governed by the scheduler’s ignition and extinction timestamps:

1. **Ignition:** instantiate from the pool, position on terrain, enable low emission; emission increases to the burning level over a configured ramp interval $T_{\text{ramp}}$.
2. **Burning:** keep flame and smoke active. Emission parameters are randomized per instance for visual diversity, and particle advection follows the current wind vector output by the meteorological component, applied as a per-particle force in the GPU particle update.
3. **Extinction:** when the extinction timestamp is reached, emission rates decay with time constant τ and particle lifetime is reduced.
4. **Residue:** disable particles, keep the burn-mark ground decal, then release particle assets back to the pool.

**Fig. 4:**
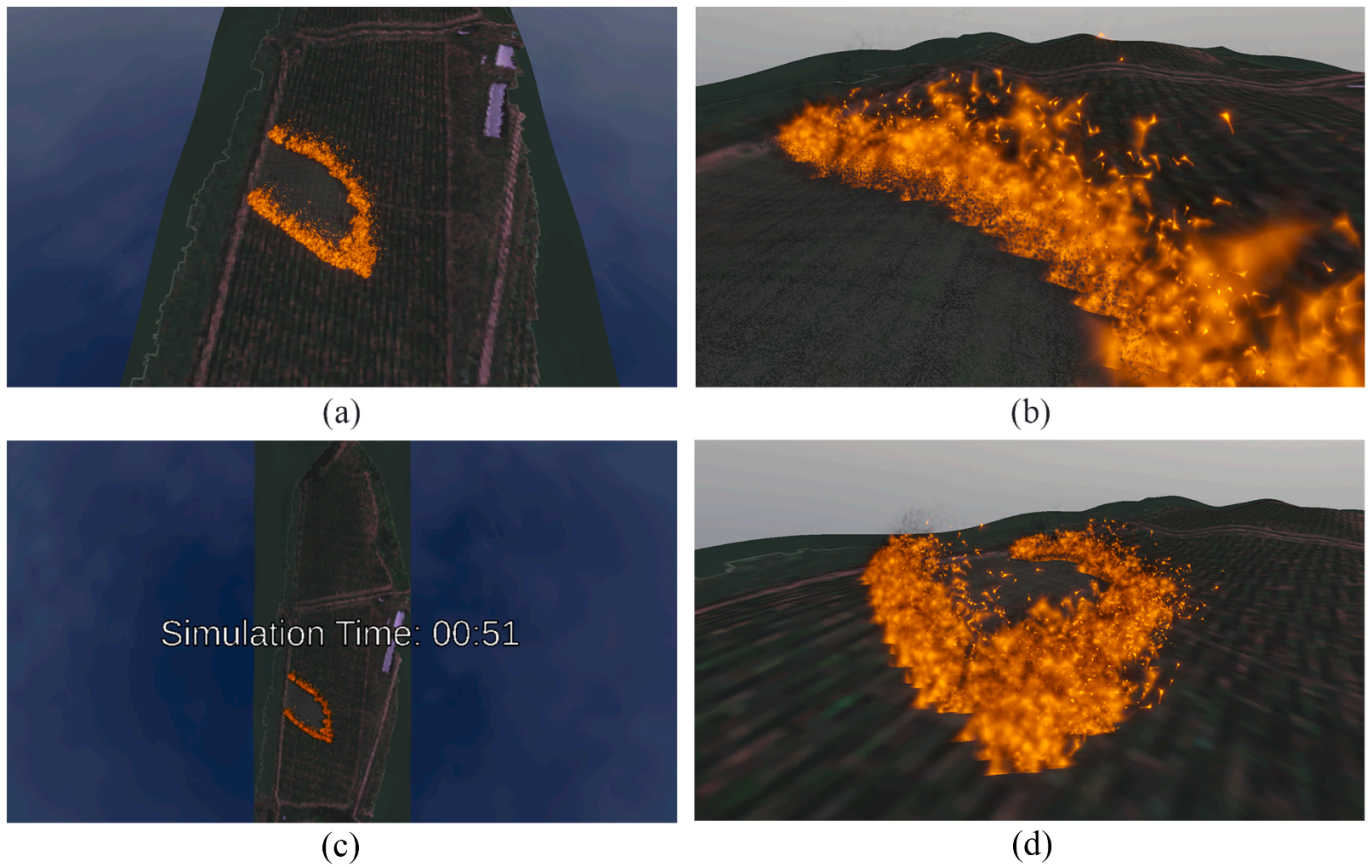
Illustrative system output: (a) overview of multiple concurrent fire events; (b) per-instance lifecycle phases; (c) temporal progression with time-coded fire events; (d) wind influence across the simulation area.

Fig. 4 illustrates the integrated rendering and data-flow pipeline in a representative configuration, showing concurrent events, lifecycle phases, temporal coding, and wind influence.

### Meteorological data integration

**Fig. 5:**
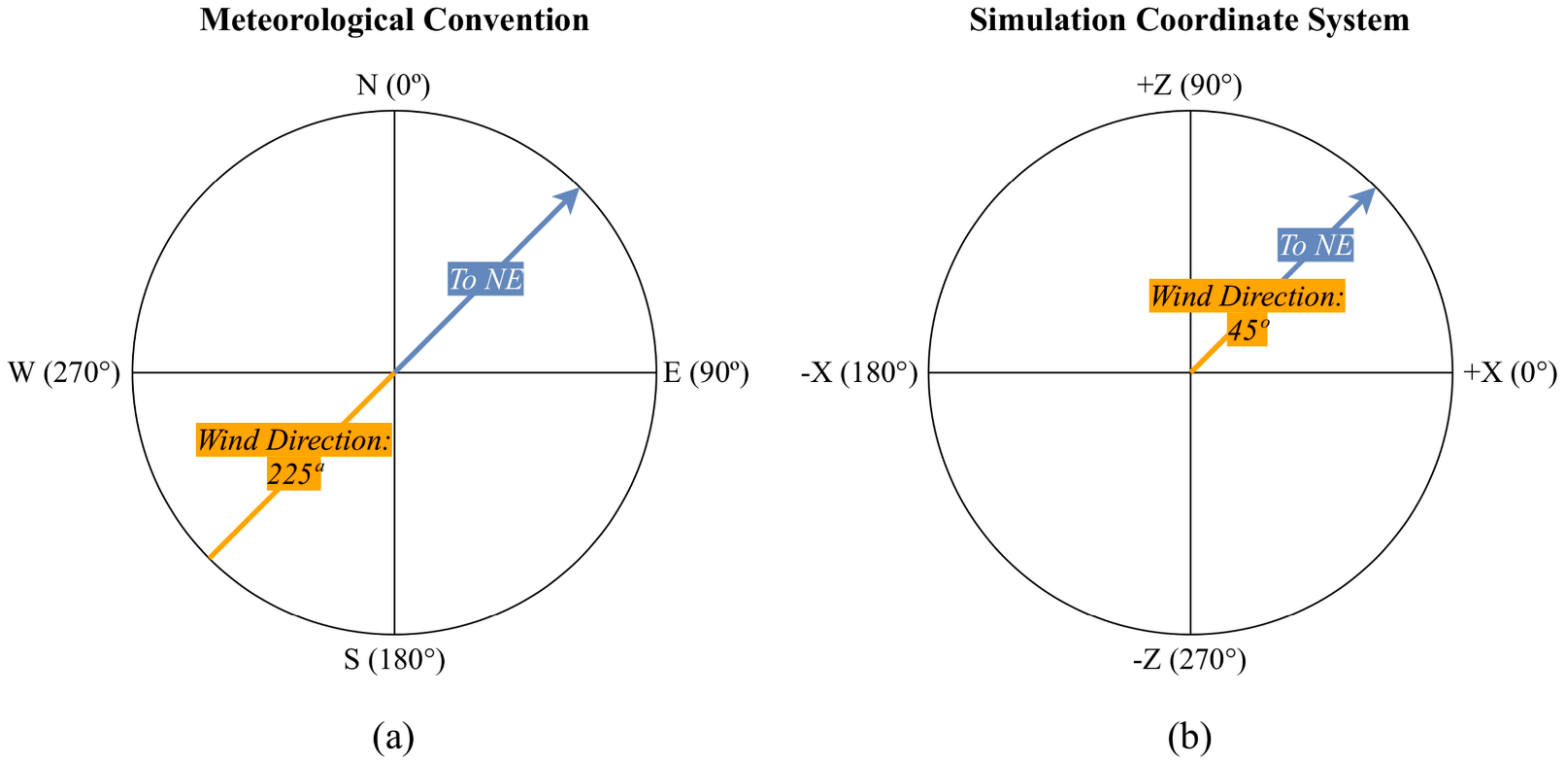
Coordinate system conversion: (a) meteorological convention measuring wind direction clockwise from North (direction *from*); (b) simulation coordinate system measuring wind direction counter-clockwise from the positive x-axis (direction *to*).

The meteorological component loads temporal wind entries containing speed and direction values at specific simulation time intervals. Each entry is converted from the meteorological convention (clockwise from North) into the simulation coordinate system using:

$$\theta_{\text{sim}}=270\circ -\theta_{\text{met}}$$

where $\theta_{\text{met}}$ represents the meteorological wind direction and $\theta_{\text{sim}}$ the corresponding simulation angle. Fig. 5 illustrates the coordinate system conversion. For example, a wind entry with direction $225^{\circ }$ (southwest) becomes $\theta_{\text{sim}}=45^{\circ }$, corresponding to northeast in the simulation. The procedure for retrieving the latest valid entry and producing the wind vector is described in Algorithm 2.

**Algorithm 2:**
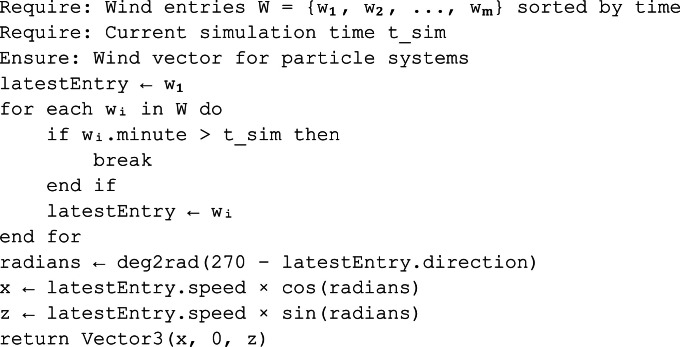
Wind vector calculation: convert time-series wind entries into a 3D vector.

**Fig. 6:**
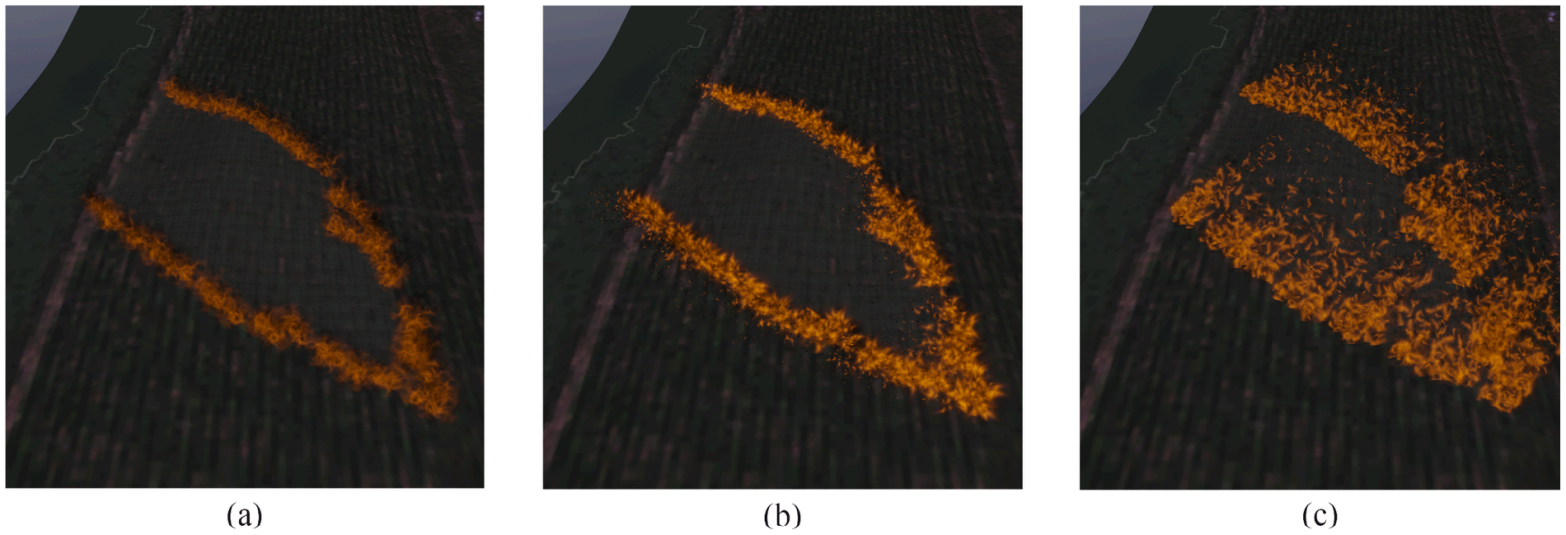
Wind influence on particle systems: (a) calm; (b) moderate wind from east; (c) strong wind from southwest.

At each simulation step, the scheduler retrieves (on the CPU) the wind entry valid for the current simulation time. Whenever the resulting vector changes, it is pushed to all active fire instances, whose GPU particle systems apply it as a per-particle advection force. Fig. 6 illustrates the resulting particle effects under different wind conditions.

### Performance optimization implementation

The proposed wildfire visualization system maintains interactive frame rates through a set of complementary performance optimizations. Object pooling, i.e., the reuse of pre-instantiated objects for visual components, reduces memory allocation overhead and minimizes garbage collection cycles. As quantified in Fig. 8, object pooling reduces frame-rate oscillations and raises the average FPS under load.

A second factor is the division of labor between CPU and GPU. Event management runs on the CPU. JSON parsing, the geodetic coordinate transformation, and terrain height sampling are executed once at load time, while the per-frame loop advances the simulation clock, activates events through the sorted list pointer, checks activated events for extinction, selects the current wind entry, and animates per-instance lifecycle parameters. The GPU, through Unity’s VFX Graph (its GPU-driven particle system) and Universal Render Pipeline, performs all per-particle computation (spawning, integration, and wind advection, with the CPU-supplied wind vector applied as a per-particle force) and all rendering, including terrain and burn-mark decals. Each active instance drives flame and smoke systems with a 500-particle capacity each, so per-particle work dominates and is fully parallelized on the GPU, consistent with the frame-time measurements in Fig. 9(b) (mean CPU 6.9 ms vs. GPU 5.2 ms on the workstation).

Memory optimization is achieved through dictionary-based caching, which stores references to frequently accessed rendering components (e.g., particle systems). This eliminates redundant per-frame component lookups in dense fire-event datasets.

Finally, the simulation loop uses indexed processing to track unprocessed fire events. The sorted list pointer ensures each event is activated only once, and lifecycle flags mark extinguished events so the per-frame checks pass over them cheaply.

## Method validation

### Evaluation methodology

The system is evaluated along two axes, spatial fidelity and performance, with results reported in the following subsections.

**Fig. 7:**
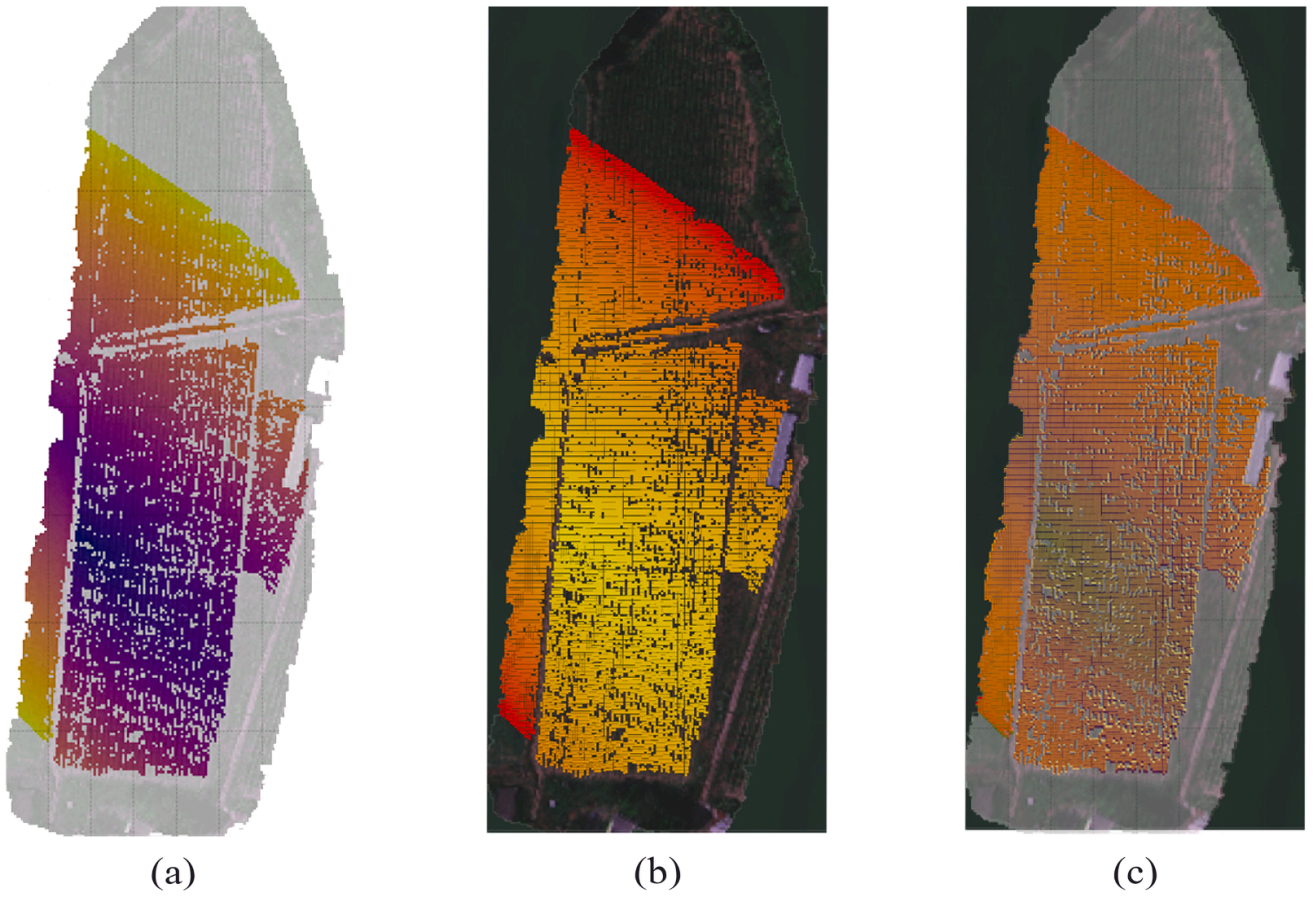
Coordinate transformation validation (**all panels are top-down**). (a) Reference ignition dataset. (b) Output of the proposed geodetic transform. (c) Overlay of (a) on (b) for visual inspection. *Color maps:* panel (a) purple → earlier, yellow → later; panel (b) yellow → earlier, red → later.

**Spatial fidelity.** Polygon and point overlays rendered on a top-down engine view allow visual inspection of the co-registration between transformed coordinates and the reference boundary and ignition points (Fig. 2, Fig. 7). In addition, spatial error is quantified per record. The coordinates produced by the implementation (single precision, as executed in the engine) are compared against double precision reference positions computed on the WGS84 ellipsoid, the standard model of Earth’s shape used for precise distance calculations, expressed in meters east and north of the domain origin. We report the mean, root-mean-square (RMSE), and maximum planar displacement.

**Performance.** The same scenario is executed twice, with identical event lists and wind trace, toggling object pooling off and on. Metrics are sampled once per second of wall-clock time over the entire simulation timeline. Simulation time runs at 1 simulation minute per real second. We report frames per second with mean and standard deviation, the active instance count over time including the observed peak and CPU and GPU frame times with mean and peak. All plots cover the full run. Metrics are collected using the engine’s profiling and frame timing instrumentation. GPU frame-time samples for which the engine’s profiler recorder returns no valid value (≈19%) are excluded from GPU statistics and plots. Hardware details are given in Performance metrics and system scalability.

### Coordinate transformation accuracy

Fig. 7 compares the reference ignition dataset with the positions produced by the geodetic transform. Panel (a) encodes ignition time in the reference dataset; panel (b) shows the positions generated by the proposed method; and panel (c) overlays (a) on top of (b) with reduced opacity for visual inspection of spatial correspondence.

For every ignition point (*n* = 18,175) and polygon vertex (*n* = 13,823), the position produced by the implementation was also compared against a double precision reference computed with geodesic calculations on the WGS84 ellipsoid. The mean planar displacement is 0.18 m (RMSE 0.21 m) with a maximum of 0.39 m, negligible relative to the size of the rendered fire effects and of the simulation domain (≈273 m × 124 m). In double precision the same transformation is accurate to below 2 mm, so the residual stems from single precision quantization of the input coordinates.

### Performance metrics and system scalability

**Fig. 8:**
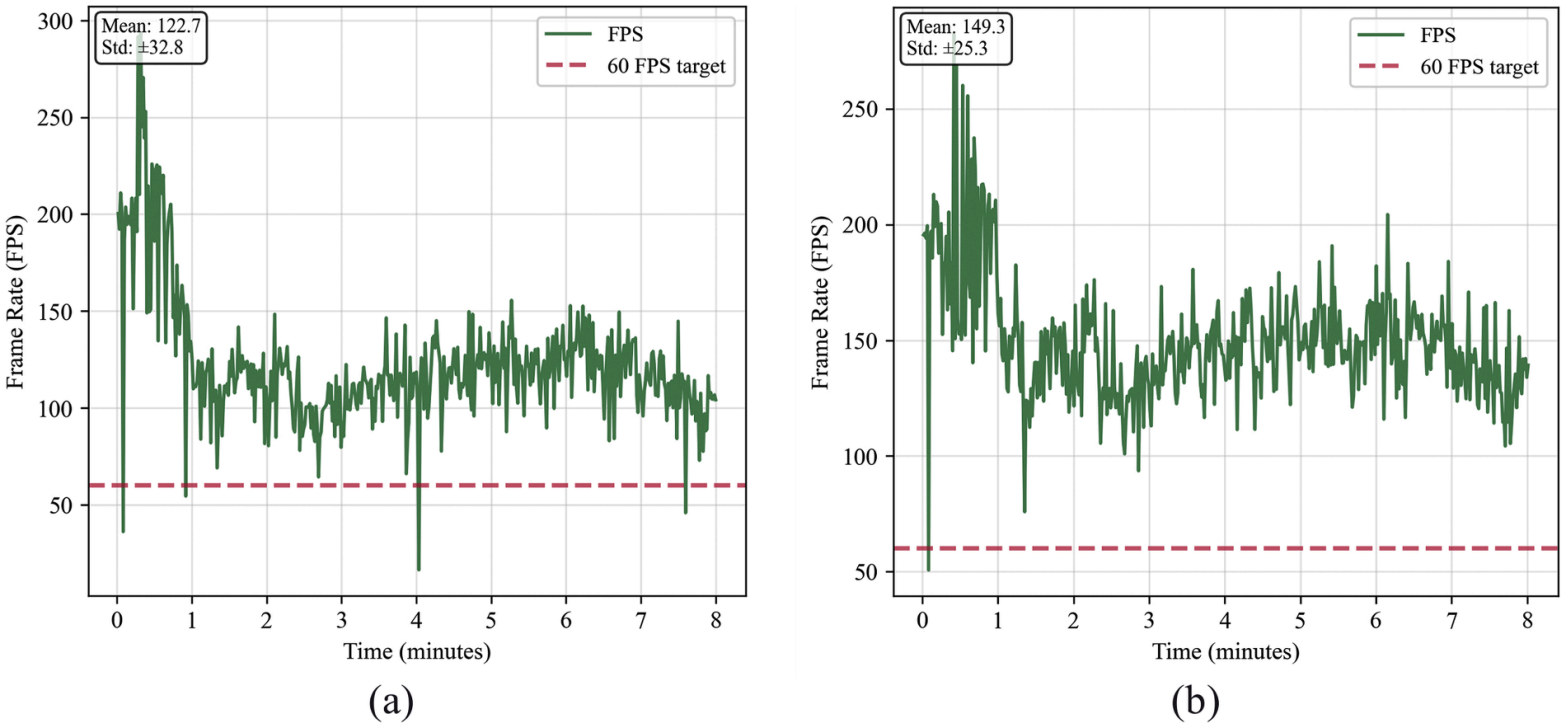
Impact of object pooling on frame-rate stability over the full simulation: (a) without pooling (mean 122.7 FPS, std 32.8); (b) with pooling (mean 149.3 FPS, std 25.3). Pooling yields a higher average frame rate and lower variability.

Fig. 8 quantifies the effect of object pooling over the full simulation. Without pooling (panel a), the frame rate averages 122.7 FPS with a standard deviation of 32.8. With pooling (panel b), the average increases to 149.3 FPS and the standard deviation decreases to 25.3. Both executions remain above the 60 FPS target, with pooling providing higher throughput and reduced variance across the simulation timeline.

Performance evaluation was performed on a workstation equipped with an NVIDIA GeForce RTX 3070, AMD Ryzen 9 7900X3D CPU, and 32 GB RAM.

**Fig. 9:**
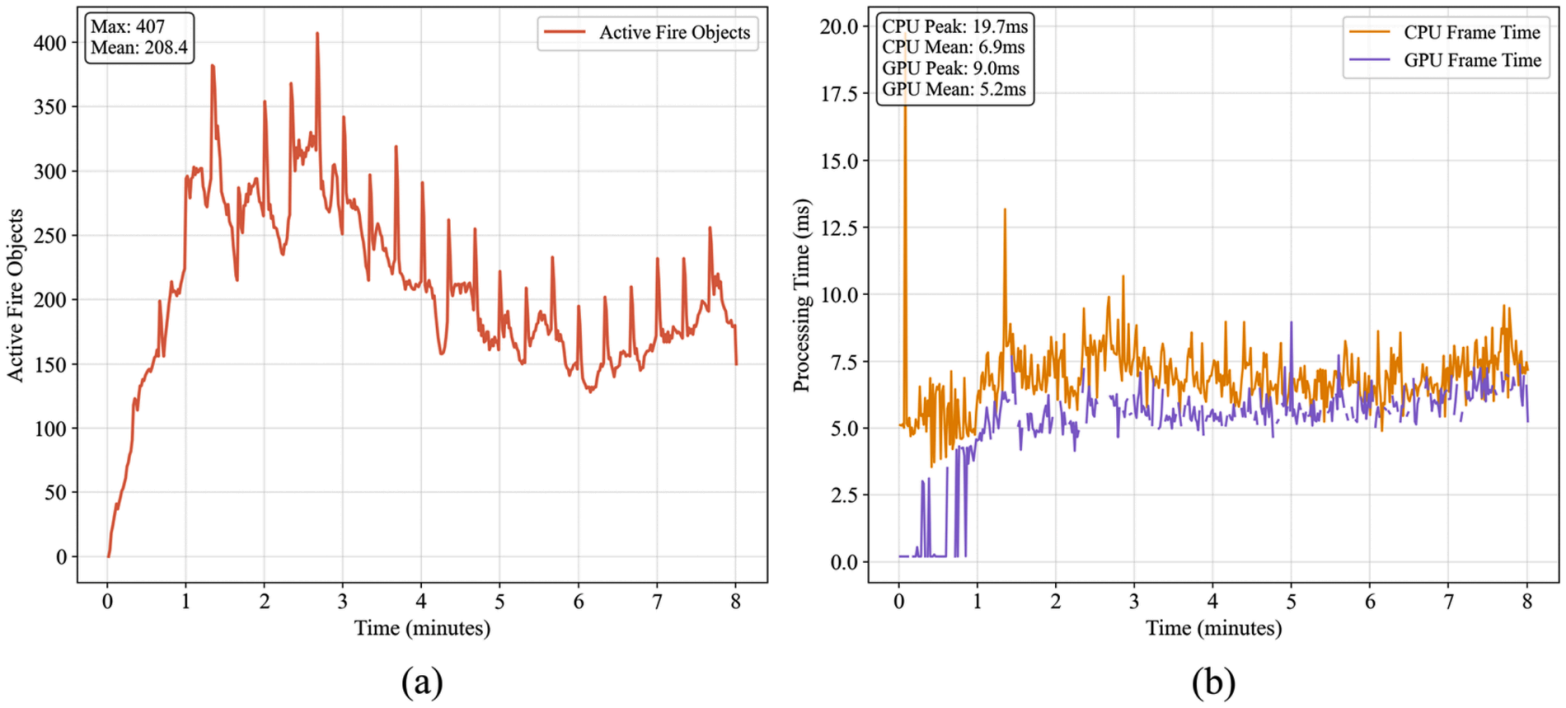
System performance metrics over the full simulation (peak = 407 active objects). (a) Active fire objects over time. (b) Frame-time profiles: CPU mean 6.9 ms (peak 19.7 ms); GPU mean 5.2 ms (peak 9.0 ms).

Fig. 9 reports active-object and frame-time profiles during the full simulation, in which the active-fire count reached a maximum of 407 objects. Panel (a) shows the active-object count over time, peaking at 407, with a mean of 208.4. Panel (b) shows CPU frame time with mean 6.9 ms (peak 19.7 ms) and GPU frame time with mean 5.2 ms (peak 9.0 ms). These values imply a CPU-limited effective frame rate of ∼145 FPS on average (1/6.9 ms) with worst-case ∼51 FPS (1/19.7 ms), i.e., above the 60 FPS target for most frames and well above the 30 FPS interactive threshold throughout.

To assess portability, the exact same simulation was also executed on a laptop equipped with an Intel Core Ultra 7 258V with integrated graphics.

**Fig. 10:**
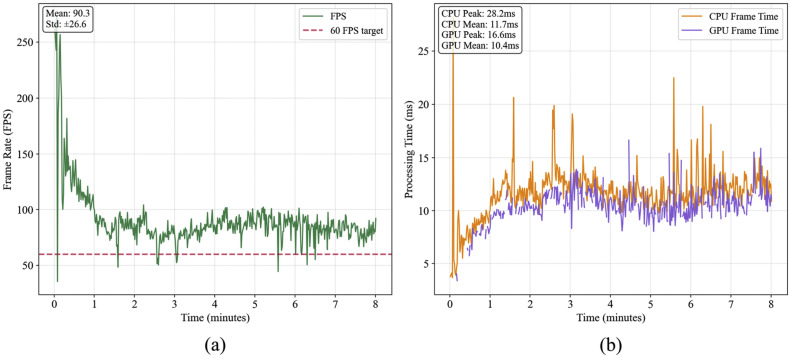
System performance metrics on a laptop with Intel Core Ultra 7 258V (integrated GPU) running the same scenario as in Fig. 8, Fig. 9. (a) Frame-rate over the full simulation (mean 90.3 FPS, std 26.6). (b) Frame-time profiles: CPU mean 11.7 ms (peak 28.2 ms); GPU mean 10.4 ms (peak 16.6 ms).

Fig. 10 shows that the mean frame rate was 90.3 FPS with a standard deviation of 26.6, remaining above the 60 FPS target for most of the run. CPU and GPU frame times were higher than on the workstation, with averages of 11.7 ms and 10.4 ms respectively, and peaks of 28.2 ms and 16.6 ms. These values correspond to an effective frame rate above 80 FPS on average, with a minimum around 36 FPS in the worst interval, remaining within interactive limits throughout the simulation.

## Limitations

The method does not model or predict fire propagation (no fuel, moisture, or rate-of-spread computation), so the fidelity of a reconstruction is bounded by the completeness and accuracy of the input event data. The current workflow requires manual preprocessing of geographic inputs, transformation fidelity depends on dataset-specific scale parameters, and the approach assumes complete and consistent ignition and extinction timestamps. Records whose extinction precedes ignition (9% of the study dataset) are extinguished immediately upon activation. Extremely large scenarios may also be bounded by GPU memory when many particle systems coexist.

## Ethics statements

This study does not involve human subjects, animal experiments, or data collected from social media platforms. No ethics approval was required.

## CRediT author statement

Rafael José: Conceptualization, Data curation, Methodology, Software, Visualization, Writing – original draft. João Carvalho: Conceptualization, Supervision, Writing – review & editing. Afonso Oliveira: Data curation, Resources, Writing – review & editing. Houda Harkat: Conceptualization, Supervision, Writing – review & editing. Nuno Fachada: Project administration, Supervision, Writing – review & editing.

## Related research article

None.

## Declaration of interests

The authors declare that they have no known competing financial interests or personal relationships that could have appeared to influence the work reported in this paper.

## Data Availability

The code and data required to reproduce the method are available in the companion repository at https://github.com/rafaeldavidjose/gpu-wildfire-visualization (MIT license). The repository includes the complete Unity project with the RealTerrain scene and all method scripts, the fire ignition and extinction event data, the fire polygon data, the wind time series, the source GeoTIFF rasters with their conversion script, and the analysis scripts behind the figures and validation tables. The fire event and polygon datasets are published with the permission of the project collaborators. The wind dataset is publicly available.

## References

[1] Huang Y., Li J., Zheng H. (2024). Modeling of wildfire digital twin: research progress in detection, simulation, and prediction techniques. Fire.

[2] Crawl D., Block J., Lin K., Altintas I. (2017). Firemap: a dynamic data-driven predictive wildfire modeling and visualization environment. Procedia Comput. Sci..

[3] Sullivan A.L. (2009). Wildland surface fire spread modelling, 1990–2007. 1: physical and quasi-physical models. Int. J. Wildland Fire.

[4] Goodchild M.F. (1992). Geographical data modeling. Comput. Geosci..

[5] Rødal S., Storli G., Gundersen O.E. (2006). Physically based simulation and visualization of fire in real-time using the graphics processing unit. Theory Pract. Comput. Graph..

[6] Castrillón-Santana M., Lorenzo-Navarro J., Hernández-Sosa D. (2011). Forecasting and visualization of wildfires in a three-dimensional geographical information system. Comput. Geosci..

[7] Wu R., Scully-Allison C., Carthen C. (2023). vFirelib: a GPU-based fire simulation and visualization tool. SoftwareX.

[8] Unity Technologies (2025). https://unity.com/.

[9] Hädrich T., Banuti D.T., Pałubicki W., Pirk S., Michels D.L. (2021). Fire in paradise: mesoscale simulation of wildfires. ACM Trans. Graph. (N.Y. NY USA).

[10] You J., Huai Y., Nie X., Chen Y. (2022). Real-time 3D visualization of forest fire spread based on tree morphology and finite state machine. Comput. Graph..

[11] Denham M.M., Waidelich S., Laneri K. (2022). Visualization and modeling of forest fire propagation in Patagonia. Environ. Model. Softw..

[12] Meng Q., Huai Y., You J., Nie X. (2023). Visualization of 3D forest fire spread based on the coupling of multiple weather factors. Comput. Graph..

[13] San Martin D., Torres C.E., Barrios C.J., Rizzi S., Meneses E., Mocskos E., Monsalve Diaz J.M., Montoya J. (2024). High Performance Computing.

[14] Kokosza A., Wrede H., Esparza D.G. (2024). Scintilla: simulating combustible vegetation for wildfires. ACM Trans. Graph. (N. Y. NY USA).

[15] Yang S., Huai Y., Nie X., Meng Q., Zhang R. (2024). Visualization of real-time forest firefighting inference and fire resource allocation simulation technology. Forests.

[16] Xia Z., Cheng S. (2025). PyTorchFire: a GPU-accelerated wildfire simulator with differentiable cellular automata. Environ. Model. Softw..

[17] Zhao Y., Ban Y. (2025). Near real-time wildfire progression mapping with VIIRS time-series and autoregressive SwinUNETR. Int. J. Appl. Earth Obs. Geoinf..

[18] Waidelich S., Laneri K.F., Denham M.M. (2018). Fire propagation visualization in real time. J. Comput. Sci. Technol..

[19] Yun S., Chen C., Li J., Tang L. (2011). Proceedings 2011 IEEE International Conference on Spatial Data Mining and Geographical Knowledge Services.

[20] Ghodrat M., Shakeriaski F., Fanaee S.A., Simeoni A. (2023). Software-based simulations of wildfire spread and wind-fire interaction. Fire.

[21] Y. Li, H. Mulyono, Y. Chen, Z. Lu, D. Chan, RtFPS: an interactive map that visualizes and predicts wildfires in the US, (2021), https://doi.org/10.48550/arXiv.2105.10880.

[22] Shennan K., Stow D.A., Nara A., Schag G.M., Riggan P. (2023). Geovisualization and analysis of landscape-level wildfire behavior using repeat pass airborne thermal infrared imagery. Fire.

[23] Sun J., Qi W., Huang Y., Xu C., Yang W. (2023). Facing the wildfire spread risk challenge: where are we now and where are we going?. Fire.

[24] Koutsaris A. (2023). Master’s thesis.

[25] Bolstad P., Manson S. (2022).

